# Preclinical Modeling of DCD Class III Donation: Paving the Way for the Increased Use of This Challenging Donor Type

**DOI:** 10.1155/2019/5924101

**Published:** 2019-09-03

**Authors:** David Soussi, Xavier Rod, Raphael Thuillier, Suzanne Leblanc, Jean-Michel Goujon, Benoit Barrou, Thierry Hauet, Thomas Kerforne

**Affiliations:** ^1^Inserm U1082, Poitiers, F-86000, France; ^2^Université de Poitiers, Faculté de Médecine et de Pharmacie, Poitiers, F-86000, France; ^3^CHU de Poitiers, Service de chirurgie générale et endocrinienne, Poitiers, F-86021, France; ^4^Groupe Hospitalier Pitié Salpétrière, Service d'Urologie et Transplantation rénale, Paris, 75013, France; ^5^CHU Poitiers, Service de Biochimie, Poitiers, F-86021, France; ^6^Service d'Anatomie Pathologique, Hôpitaux Civils de Colmar, 39 avenue de la liberté 68024 Colmar, France; ^7^CHU de Poitiers, Service d'Anatomie et Cytologie Pathologique, Poitiers, F-86021, France; ^8^Fédération Hospitalo-Universitaire SUPORT, Poitiers, F-86000, France; ^9^IBiSA Plateforme ‘plate-forme MOdélisation Préclinique-Innovation Chirurgicale et Technologique (MOPICT)', Domaine Expérimental du Magneraud, Surgères, F-17700, France; ^10^CHU Poitiers, Service de Réanimation Chirurgie Cardio-Thoracique et Vasculaire, Coordination des P.M.O., Poitiers, F-86021, France

## Abstract

Deceased after circulatory death (DCD) donors offer a viable solution to the current organ shortage, particularly the Maastricht Class III (arrest subsequent to cessation of life support in the hospital). Although current results from these donors are very satisfactory, the number of included donors is too low and future expansion of inclusion criteria will likely decrease organ quality, with negative consequences on the complication rate. This donor type thus represents a priority in terms of scientific exploration, so as to study it in controlled settings and prepare for future challenges. Hence, we mimicked the DCD Class III clinical conditions a Large White pig model. Herein, we detail the different strategies attempted to attain our objectives, including technical approaches such as animal positioning and ventilator settings, as well as pharmacological intervention to modulate blood pressure and heart rate. We highlight the best combination of factors to successfully reproduce DCD Class III conditions, with perfusion pressures and functional warm ischemia (hypoperfusion) closely resembling clinical situations. Finally, we detail the functional and histological impacts of these conditions. Such a model could be of critical value to explore novel management alternative for these donors, presenting a uniquely adapted platform for such therapeutics as normothermic regional circulation and/or pharmacological intervention.

## 1. Introduction

Currently, kidney transplantation is the best therapy to treat chronic renal failure, both in terms of survival and in terms of quality of life for the patients, as well as in terms of public health [1–3].

In 2017 in France, the regulatory agency Agence de la Biomedicine (ABM) [4] recorded 3782 kidney transplantations, a 30% increase compared to 2008. This increase was made possible through the extension of donor acceptance criteria, increased living donation, as well as the improvement of immunosuppressive and preservation techniques, for instance, using machine perfusion. Unfortunately, these measures were unable to match the growing expansion of the waiting list, which numbered 18793 patients in 2017, a never before reached level of shortage.

To face this, the ABM mounted a vast program [5], with such priorities as new preservation strategies and particularly development of new organ procurement conditions. Among these, donors deceased by circulatory death (DCD) are at the forefront.

These donors are categorized following the Maastricht classification [6], among which two classes are of interest in transplantation:

(i) Class II: patients in circulatory arrest with rapid cardiac massage and mechanical ventilation maneuvers were put in place but unable to recover circulatory activity. The first French transplantation from a class II donor was performed in Lyon in 2006, followed by 42 more in 7 centers, exclusively with kidneys. After evaluation, the ABM determined the initiative to the valid and extended the authorization to the liver in 2010. However, although representing a significant advance in terms of technique and ethics, only 120 donors are reported each year, allowing approximately 60 kidney and 2 to 5 hepatic transplantations. This number is unlikely to increase due to the technical and logistical difficulties inherent to this donor type. This led to the consideration of other Maastricht categories.

(ii) Class III: patients for which the decision to cease life support is programmed due to the diagnostic considerations. While this class represented more than 90% of DCDs worldwide, France waited until 2014 to allow initial trials, mostly due to ethical considerations. Since 2015, 102 procedures have been successfully completed with 182 kidney and 50 hepatic transplantation performed. The objective is to increase the use of this category to a minimum of 500 by 2021, which will require significant improvement of techniques and care, as well as extending the donor acceptance criteria.

Such objectives, and the likely further extension of Class III DCD use in years to come, require a proper modelization of this situation in animals. Indeed, while current procedures are safely conducted and results are very encouraging, these are performed on ‘ideal' donors which cannot realistically meet the demand for organs. Similarly to what was decided for brain death donors, it is likely that extension of donor criteria for Class III DCD will take place in the future. The difficulties which will stem from such decisions will need to be anticipated as much as possible if the community wants to avoid the drawbacks encountered after the extended criteria brain death donor classification was accepted, with increased rates of complications such as delayed graft function and shorter graft survival.

Herein, we describe a new large animal model of DCD Class III donor, mimicking as close to possible the situation encountered in the intensive care units. Strategies considered and tested are presented and discussed, and the final version is described in details and completed with biological, histopathological and biochemical evaluations.

## 2. Materials and Methods

### 2.1. Animals

Animal experiments were conducted at the MOPICT platform (Surgères, France) in accordance with the ARRIVE guidelines, the French Government and the institutional Committee on the Ethics of Animal Experiments (France) (Committee accreditation number: C2EA-84, Protocol approval number: CE2012-11). We used 3-month-old Large White male pigs (40-45kg), a pertinent preclinical model, as its anatomy and physiology are very close to humans [7].

### 2.2. Anesthesia

Anesthetic induction was performed with a Hunter mask with a mixture of oxygen associated with 8% sevoflurane. The animal was monitored with cardioscope and perfused at a marginal vein of the ear. A central venous catheter was positioned in the internal jugular vein after right cervicotomy. Further anesthesia was followed by Propofol, Fentanyl, Ketamin, and Rocuronium. Pain management was performed with Fentanyl.

Further procedures are integrated to the description of the modelization process, hence detailed in the Results section.

At the end of the protocol, euthanasia was accomplished by injection of a lethal dose of KCl and confirmed by flat electrocardiogram and two-channel encephalogram.

### 2.3. Blood Parameters Evaluation and Histologic Evaluation of Kidney Cortex at the End of the M III Protocol

Plasma and urine biochemistry parameters were measured using a Cobas bioanalyser (Roche-Diagnostics, Meylan, France). Urine Neutrophil Gelatinase-Associated Lipocalin (NGAL), plasma Tumor Necrosis Factor alpha (TNF*α*) and high–mobility group box 1 (HMGb1) were determined by ELISA immunoassay according to the manufacturer's instructions (R&D Systems, Abingdon, UK). Histologic evaluation of kidney cortex was performed at the end of the MIII protocol. Briefly, two pathologists evaluated separatly typical kidney lesions in a blind manner following a semi-quantitative scale. For leukocytes infiltrating renal interstitium: 0: none; 1: light, localized; 2: light, diffuse; 3: intense, localized; 4: intense, diffuse. For Tubular dilation, brush border loss and necrosis: 0: none; 1: < 25%; 2: 25-50%; 3: 50-75%; 4: > 75%.

### 2.4. Statistical Analysis

The results are expressed either in dot plots with individual data point as well as mean±SD as a line. For statistical analysis, the Friedman test was used as the non-parametric equivalent of the repeated measure ANOVA; this test includes a post hoc multiple comparison test between time points. R software was used; statistical significance was fixed at p<0.05.

## 3. Modelization Processes and Results

### 3.1. Current Clinical Class III Parameters

**Figure 1:**
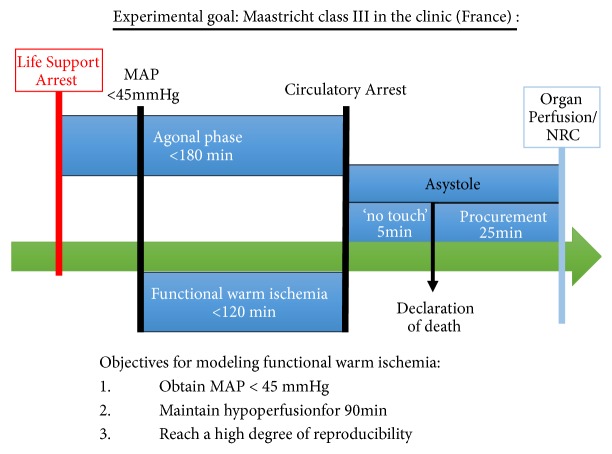
*Schematic representation of the DCD ClassIII guidelines formulated by the ABM:* Agonal period (between life support cessation and circulatory arrest) must last under 180min, and within this time window the hypoperfusion time (i.e., between the point when mean arterial pressure, MAP, drops below 45 mmHg and circulatory arrest) must be under 120min. Once circulatory arrest is determined, there is a 5min no touch period before death is declared and organs can be procured. Further management considerations such as normothermic regional circulation are currently being discussed.

The main limitation of DCD Class III resides in the delay between the start of the life support cessation and the circulatory arrest. Indeed, while hemodynamic conditions deteriorate rapidly, hours can pass before arrest. In this light, the ABM established strict guidelines and time limitations to define DCD Class III acceptance criteria (Figure 1): the agonal (between life support cessation and circulatory arrest) must last under 180min, and within this time window the hypoperfusion time (i.e., between the point when mean arterial pressure, MAP, drops below 45 mmHg and circulatory arrest) must be under 120min. Once circulatory arrest is determined, there is a 5min no touch period before death is declared and organs can be procured. Further management considerations such as normothermic regional circulation [8] are currently being discussed.

Taking these parameters into account, the objectives of our models were defined as:

1. Obtain a MAP inferior to 45mmHg
2. Maintain this hypoperfusion for at least 90min
3. Reach a high degree of reproducibility.

### 3.2. Reaching the MAP and Duration Objectives

According to the hemodynamic Ohm's law, mean arterial pressure (MAP) determinants are the cardiac output (CO), the central venous pressure (CVP) and the systemic vascular resistance (SVR) following the equation: MAP – CVP = CO x SVR

The determinants of cardiac output are the heart rate (HR) and the stroke volume (SV) according to the following: CO = HR x SV

Hence combining the two equations we obtain: MAP – CVP = HR x SV x SVR

Thus, decreasing MAP requires the decrease of cardiac output through diminution of heart rate and/or SV and/or SVR. The first option could have been the controlled decrease in blood volume, which would have decreased cardiac output through SV diminution. However, lower blood volume would promote ischemia reperfusion lesions and not be comparable to the situation in the clinic. Reproducibility could also have been problematic. We thus adopted a pharmacological and mechanical strategy. Such strategy must include the following:

- An inotropic effect to decrease SV
- A negative chronotropic effect to diminish HR
- An arterial vasodilatation effect to decrease SVR

Finally, the pharmacokinetic properties had to permit a fast delay of action and a short duration of effect in order to manage an in adapted response.

With these specifications in mind, several options were selected:

(i) Esmolol/Brevibloc, an i.v. beta blocker with a short delay and duration of action with a negative inotropic and chronotropic effect but low vasoplegic properties. It was tested at 125 *μ*g/kg/min with a syringe pump, with an increase in the flow of 50*μ*g/kg/h every 10 minutes.

(ii) Atenolol/Tenormin, an i.v. beta blocker with a short delay and duration of action with a negative inotropic and chronotropic effect as well as arterial vasoplegic properties. It was tested at 20 *μ*g/kg/min with a syringe pump, with an increase in the flow of 10*μ*g/kg/h every 5 minutes.

(iii) Nicardipine/Loxen, an i.v. calcic inhibitor with a short delay and duration of action as well as arterial vasoplegic properties but inducing a reflex tachycardia. It was tested at 100 *μ*g/kg/min with a syringe pump, with 100*μ*g/kg/h boluses and an increase in the flow of 25*μ*g/kg/h every 5 minutes.

Each was tested in the pig. It was rapidly evidenced that the beta blockers were not effective in this animal: their alpha and beta blocking properties were insufficient on vasoplegia, inotropism and chronotropism. We thus chose to use Nicardipine, which had sufficient effects on vasoplegia to reach a target diastolic pressure of 30 mmHg but induced an important reflex tachycardia, allowing the animal to maintain an important cardiac output and pulsed arterial pressure due to the absence of negative inotropic effect of the molecule. We thus associated a negative chronotropic agent with was not a beta blocker. We chose Lidocain/Xylocaine which possess this effect, injected as soon as the heart rate was above 220 beats per minute and as long as MAP remained above 45 mmHg at a dose of 4mg/kg every 5min until the MAP goal was attained.

Furthermore, reduction of the venous return was obtained through mechanical strategies: first, the animal was positioned at 15° declivity, and then the ventilation was altered to mimic hypoventilation observed after extubating in the clinic: expiratory pressure was increased at 10 cm H2O and could be further increased to 20cm in case the blood pressure response was insufficient; moreover, ventilation rate was halved in parallel with the respiratory frequency to obtain a current volume of 10mL/kg; finally, FiO2 was decreased to 40%.

In case of unwanted arterial blood response, expiratory pressure was decreased to 5 cm H2O and the base Nicardipine flow to 100 *μ*g/kg/h. Ephedrin at 6mg i.v. could be added and the Nicardipin stopped in case of persisting low blood pressure.

To further mimic the clinical protocol as codified by the ABM, heparin was used at a dose of 100 U/kg i.v. This was aimed at decreasing clot formation during the hypo circulation phase. In our hands, this was administered when PAM was stably under 45 mmHg.

**Figure 2:**
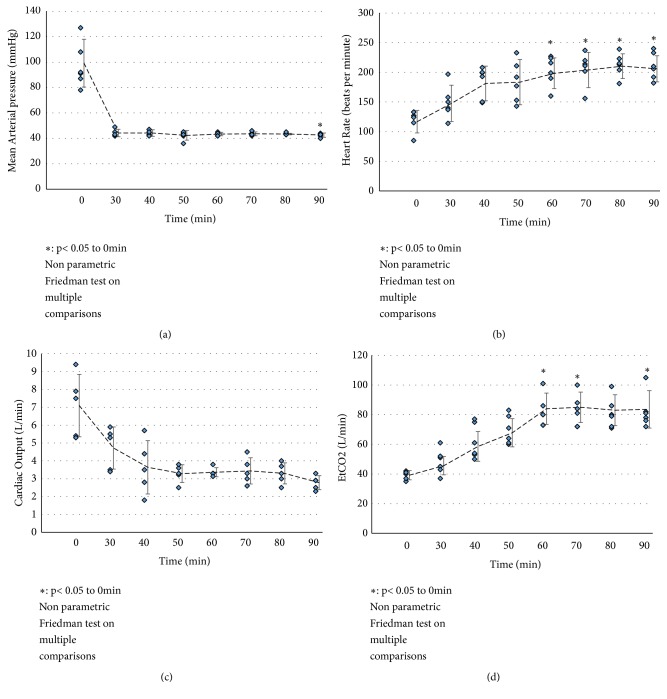
*Donor surveillance parameters: *Pigs were followed during the protocol for key parameters in the DCD Class III definition. (a) Mean Arterial pressure; (b) Heart Rate; (c) Cardiac Output; (d) EtCO2. Shown are individual data points as well as mean±SD. Statistics were performed with the Friedman test. Overall p value in the Friedman test was 0.014 for MAP, <0.001 for Heart Rate, 0.012 for Cardiac output, and <0.001 for EtCO2.

Using this protocol, we were able to reach a MAP inferior to 45mmHg as early as 30 min after the start of the protocol (Figure 2(a)). This involved an increase in heart rate to 200 beats per minute after 40-50 min (Figure 2(b)), a rapid decrease of cardiac output (approximately 60% in 50 min, remaining stable afterwards, Figure 2(c)) and significant hypercapnia (reaching 80 L/min, Figure 2(d)).

### 3.3. Biological, Histopathological and Molecular Surveillance

**Figure 3:**
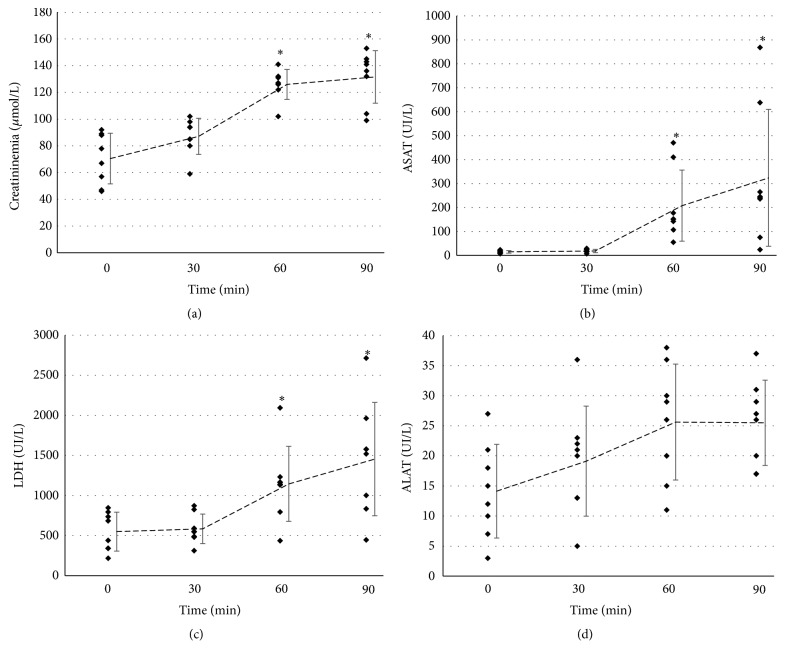
*Biological parameters and circulating markers of injury*: Pigs were followed during the protocol for biochemical parameters. (a) Serum creatinine; (b) Aspartate Aminotransferase; (c) Lactate dehydrogenase; (d) Alanine Aminotransferase. Shown are individual data points as well as mean±SD. Statistics were performed with the Friedman test. Overall p value in the Friedman test was <0.001 for creatininemia, <0.001 for LDH, <0.001 for ASAT and 0.062 for ALAT.

We recorded a regular increase in creatininemia as soon as the protocol started, reaching 120*μ*mol/L by 60 min (30 min after MAP went under 45mmHg) indicating that kidney function was impacted (Figure 3(a)). Estimating the impact on necrosis, we measured systemic Aspartate Amino Transferase (ASAT) which only increased after MAP goal was reached but rapidly reached 200-400 UI/L; circulating lactate dehydrogenase (LDH) was also increased after MAP reached under 45 mmHg but significantly doubled and tripled (Figure 3(c)); finally we detected increased levels of Alanine Amino Transferase (ALAT), specific of kidney cytolysis, but they remained very low and not significant.

**Figure 4:**
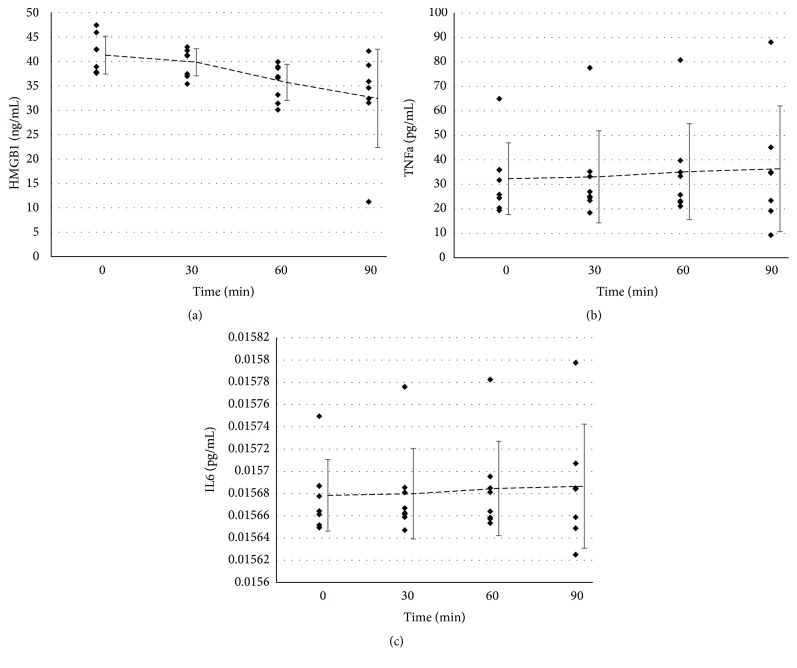
*Circulating markers of inflammation*: Pigs were followed during the protocol for inflammation markers. (a) High–Mobility Group box 1; (b) Tumor Necrosis Factor *α*; (c) Interleukin 6. Shown are individual data points as well as mean±SD. Statistics were performed with the Friedman test.

To monitor the systemic inflammatory state, we measured the presence of proinflammatory molecules in the plasma. The alarmin HMGB1 actually tended to decrease over time, without reaching significance (Figure 4(a)), and similarly the cytokines TNF*α* (Figure 4(b)) and IL-6 (Figure 4(c)) did not show alteration of their circulating level.

**Figure 5:**
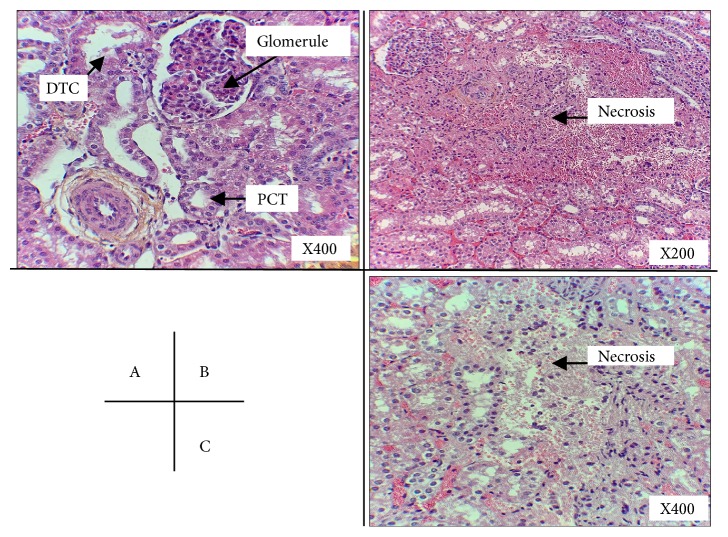
*Anatomopathological evaluation of kidney histology at the end of MIII protocol:* Representative images of histology are presented. A: HE coloration, renal cortex: normal tissue (From Animal I; Magnification 400X), DCT: distal convoluted tubule, PCT: proximal convoluted tubule; B: HE coloration, renal cortex: foci of necrosis (From Animal VI, Magnification: 200X) C: HE coloration, renal cortex: foci of necrosis (From Animal VI, Magnification: 400X).

Histologic evaluation of kidney cortex at the end of the protocol did not detect significant increase in typical ischemia reperfusion lesions such as lymphocyte infiltration, tubular dilation, brush border loss and necrosis (Table 1, Figure 5).

## 4. Discussion

Herein we describe a protocol to mimic the conditions of donation after circulatory death, Class III, in a preclinical model using the pig. Through a combination of pharmacological and technical approaches, we reliably decrease MAP to below 45mmHg and maintain the animal in this state for 90 minutes. Heparin is also added as soon as MAP objectives are reach to follow the ABM guidelines.

Our model is not the first to pretend approximating Class III DCD conditions:

(i) A first approach stops ventilation support while anesthetics are still administered. In one case [9], functional warm ischemia lasted 15 min followed by 15min warm ischemia; in another case [10] death occurred between 15 and 20 minutes.

(ii) Another strategy proposed a lethal injection of potassium followed by a period of warm ischemia lasting between 45 in mice [11] and 60 min in pig [12], even reaching 150min which is closer to the timing of Class III DCD.

(iii) Studying the kidney, mimicking DCD condition was attempted by clamping of the renal pedicule [13], which in our own experience reliably increase the level of ischemia reperfusion injury [14, 15].

These approaches are however largely insufficient to properly evaluate secondary kidney lesions due to prolonged hypoperfusion, as found in humans. Indeed in these models the agonal phase rarely lasts beyond 30min, while the ABM guidelines authorizes 120 min of functional warm ischemia. We indeed believe that current guidelines present an important risk for the kidney. Its function include maintenance of homeostasis through hydric, acido-basic and arterial pressure control; production of renin, erythropoietin and 1,25 vitamin D, participating in neoglucogenesis using amino acid and lactate, and finally the elimination of organic refuse (urea, creatinine, uric acid…) and exogenous chemicals (toxics, drugs…). These activities are highly dependent on kidney perfusion, and thus MAP. Between 80 and 150 mmHg, arterial pressure permits a maintenance of glomerular filtration rate (GFR) through a feedback loop between afferent and efferent arterioles. However, below 80mmHg, GFR falls rapidly and the nephron cannot function.

In our hands, the gradual increase in cytolysis markers LDH and ASAT indicates parenchymal lesions building as soon as hypoperfusion is attained. Since ALAT is unaffected, it is likely that these lesions do not involve the liver. It thus appear that our protocol reliably impacts the kidney, although this could not be confirmed by histopathological evaluation.

As this is not surprising due to the limitation of this technique, particularly in detecting light lesions, we performed further analysis on the kidney cortex proteins involved in lesion pathways (Supplementary figures ([Supplementary-material supplementary-material-1])) and our data suggested: a- increased response to hypoxia (through EPO production); b-fostering of a pro-coagulation milieu (increased Tissue Factor and its activation); c- decreased vascular reactivity (lower eNOS levels); d- a proinflammatory milieu (increase ICAM production). This suggests that while not in itself capable of inducing observable lesions to the kidney, our protocol created a pro-injury milieu in the organ which will likely be more sensitive to ischemia reperfusion lesions encountered as a result of organ transplantation.

## 5. Conclusion

The ever growing organ shortage imposes the need for new sources of organs, with donors likely becoming less ideal providing organ with low quality. The DCD Class III donor represents a high number of organs in the near future, and will likely see its acceptance criteria widened. To anticipate the difficulties that are certain arise from this dynamic, we developed a porcine model of DCD Class III, which closely mimics the situation in the clinic. We demonstrate that indeed the donor management procedures have an impact on the kidney, creating unstable conditions prior to procurement and transplantation, hence likely fostering a higher rate of complications.

This model is perfectly suited to studying new donor management methods and therapeutics aimed at preserving the organs.

## Figures and Tables

**Table 1:** Anatomopathological evaluation of kidney histology at the end of MIII protocol (90 min).

Animal	Leukocytes Infiltrate	Tubular Dilation	Brush Border Loss	Necrosis
I	0	0	0	0
II	1	0	0	0
III	0	0	0	0
IV	0	0	0	0
V	0	0	0	0
VI	0	0	1	1
VII	0	0	0	0
VIII	0	0	1	1

Kidney cortex samples were collected at the end of the MIII protocol and processed for histological evaluation. A blind pathologist evaluated typical kidney lesions following a semi-quantitative scale. For leukocytes infiltration: 0: none; 1: light, localized; 2: light, diffuse; 3: intense, localized; 4: intense, diffuse. For Tubular dilation, brush border loss and necrosis: 0: none; 1: < 25%; 2: 25-50%; 3: 50-75%; 4: > 75%.

## Data Availability

The data used to support the findings of this study are available from the corresponding author upon request.

## Supplementary Materials

Supplementary Materials Supplementary Figure 1 Protein quantification: Western Blotting was carried out according to standard protocols using specific antibodies against: HIF-1*α*, EPO (BD Transduction-Laboratories, Pont de Claix, france), eNOS total and phophorylated Ser1177, Bax and Bcl2 (Cell-Signaling, Saint Quentin-Yvelines, France), Thrombomodulin, total and phophorylated Tissue-Factor (Abcam, Cambridge, United-Kingdom), and InterCellular Adhesion Molecule-1 (ICAM-1) and VCAM-1 (Bioss, Woburn, Massachusetts, USA), E-Selectin and loading control *β*-actin (Sigma-Aldrich, Lyon, France). Each individual staining is presented as follow:* Top:* Stain Free gel for normalization,* Middle*: full image of the western blot,* Bottom*: quantification of the indicated (red square) bands, normalized to the stain free gel.* Top Panel*: A: HIF1alpha; B: Thrombomodulin; C: Bcl2; D: Bax.* Middle Panel*: E: Tissue Factor; F: Phosphorylated Tissue Factor; G: eNOS; H: Phosphorylated eNOS.* Bottom Panel*: I: VCAM; J: ICAM; K: EPO.
