# Supplementary material for: Preclinical Modeling of DCD Class III Donation: Paving the Way for the Increased Use of This Challenging Donor Type
Source: Biomed Res Int. 2019 Sep 3;2019:5924101. doi: 10.1155/2019/5924101 (PMC6745153; doi:10.1155/2019/5924101)
Supplement: Supplementary Materials — Supplementary Figure 1 Protein quantification: Western Blotting was carried out according to standard protocols using specific antibodies against: HIF-1α, EPO (BD Transduction-Laboratories, Pont de Claix, france), eNOS total and phophorylated Ser1177, Bax and Bcl2 (Cell-Signaling, Saint Quentin-Yvelines, France), Thrombomodulin, total and phophorylated Tissue-Factor (Abcam, Cambridge, United-Kingdom), and InterCellular Adhesion Molecule-1 (ICAM-1) and VCAM-1 (Bioss, Woburn, Massachusetts, USA), E-Selectin and loading control β-actin (Sigma-Aldrich, Lyon, France). Each individual staining is presented as follow: Top: Stain Free gel for normalization, Middle: full image of the western blot, Bottom: quantification of the indicated (red square) bands, normalized to the stain free gel. Top Panel: A: HIF1alpha; B: Thrombomodulin; C: Bcl2; D: Bax. Middle Panel: E: Tissue Factor; F: Phosphorylated Tissue Factor; G: eNOS; H: Phosphorylated eNOS. Bottom Panel: I: VCAM; J: ICAM; K: EPO. [file 5924101.f1.pptx]

## Slide 1
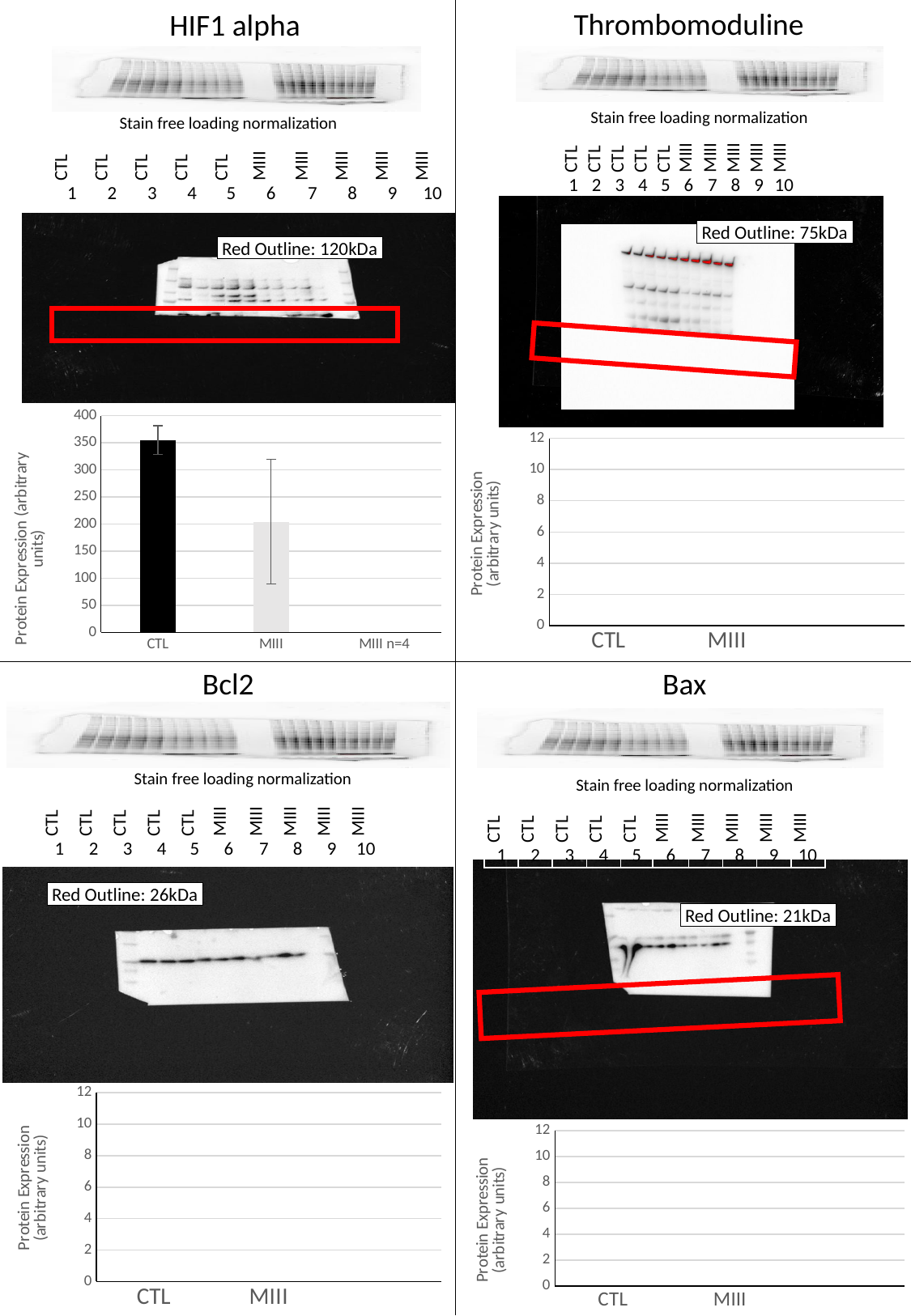

HIF1 alpha
Thrombomoduline
Stain free loading normalization
Stain free loading normalization
| CTL | CTL | CTL | CTL | CTL | MIII | MIII | MIII | MIII | MIII |
| --- | --- | --- | --- | --- | --- | --- | --- | --- | --- |
| 1 | 2 | 3 | 4 | 5 | 6 | 7 | 8 | 9 | 10 |
| CTL | CTL | CTL | CTL | CTL | MIII | MIII | MIII | MIII | MIII |
| --- | --- | --- | --- | --- | --- | --- | --- | --- | --- |
| 1 | 2 | 3 | 4 | 5 | 6 | 7 | 8 | 9 | 10 |
Red Outline: 75kDa
Red Outline: 120kDa
[unsupported chart]
### Chart
| Category | |
|---|---|
| CTL | 344.62325999999996 |
| MIII | 392.31626 |Bcl2
Bax
Stain free loading normalization
Stain free loading normalization
| CTL | CTL | CTL | CTL | CTL | MIII | MIII | MIII | MIII | MIII |
| --- | --- | --- | --- | --- | --- | --- | --- | --- | --- |
| 1 | 2 | 3 | 4 | 5 | 6 | 7 | 8 | 9 | 10 |
| CTL | CTL | CTL | CTL | CTL | MIII | MIII | MIII | MIII | MIII |
| --- | --- | --- | --- | --- | --- | --- | --- | --- | --- |
| 1 | 2 | 3 | 4 | 5 | 6 | 7 | 8 | 9 | 10 |
Red Outline: 26kDa
Red Outline: 21kDa
### Chart
| Category | |
|---|---|
| CTL | 145.52788 |
| MIII | 145.664602 |
### Chart
| Category | |
|---|---|
| CTL | 476.5340666666666 |
| MIII | 377.71655999999996 |

## Slide 2
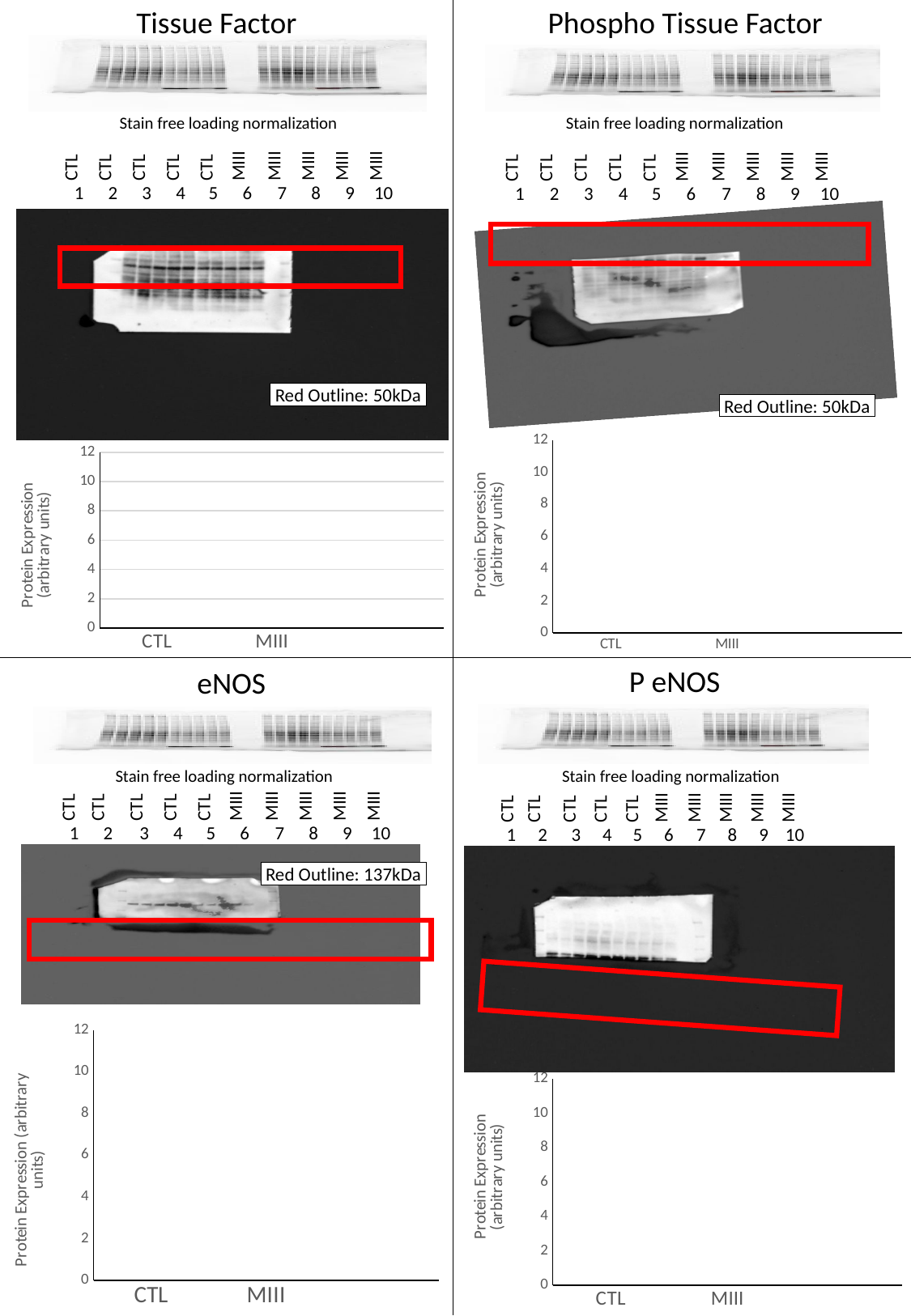

Tissue Factor
Phospho Tissue Factor
Stain free loading normalization
Stain free loading normalization
| CTL | CTL | CTL | CTL | CTL | MIII | MIII | MIII | MIII | MIII |
| --- | --- | --- | --- | --- | --- | --- | --- | --- | --- |
| 1 | 2 | 3 | 4 | 5 | 6 | 7 | 8 | 9 | 10 |
| CTL | CTL | CTL | CTL | CTL | MIII | MIII | MIII | MIII | MIII |
| --- | --- | --- | --- | --- | --- | --- | --- | --- | --- |
| 1 | 2 | 3 | 4 | 5 | 6 | 7 | 8 | 9 | 10 |
Red Outline: 50kDa
Red Outline: 50kDa
### Chart
| Category | |
|---|---|
| CTL | 368.51166 |
| MIII | 596.5554199999999 |
### Chart
| Category | |
|---|---|
| CTL | 47.502337999999995 |
| MIII | 60.102610000000006 |P eNOS
eNOS
Stain free loading normalization
Stain free loading normalization
| CTL | CTL | CTL | CTL | CTL | MIII | MIII | MIII | MIII | MIII |
| --- | --- | --- | --- | --- | --- | --- | --- | --- | --- |
| 1 | 2 | 3 | 4 | 5 | 6 | 7 | 8 | 9 | 10 |
| CTL | CTL | CTL | CTL | CTL | MIII | MIII | MIII | MIII | MIII |
| --- | --- | --- | --- | --- | --- | --- | --- | --- | --- |
| 1 | 2 | 3 | 4 | 5 | 6 | 7 | 8 | 9 | 10 |
Red Outline: 137kDa
### Chart
| Category | |
|---|---|
| CTL | 656.27634 |
| MIII | 363.00057499999997 |
### Chart
| Category | |
|---|---|
| CTL | 87.33977999999999 |
| MIII | 75.65345 |

## Slide 3
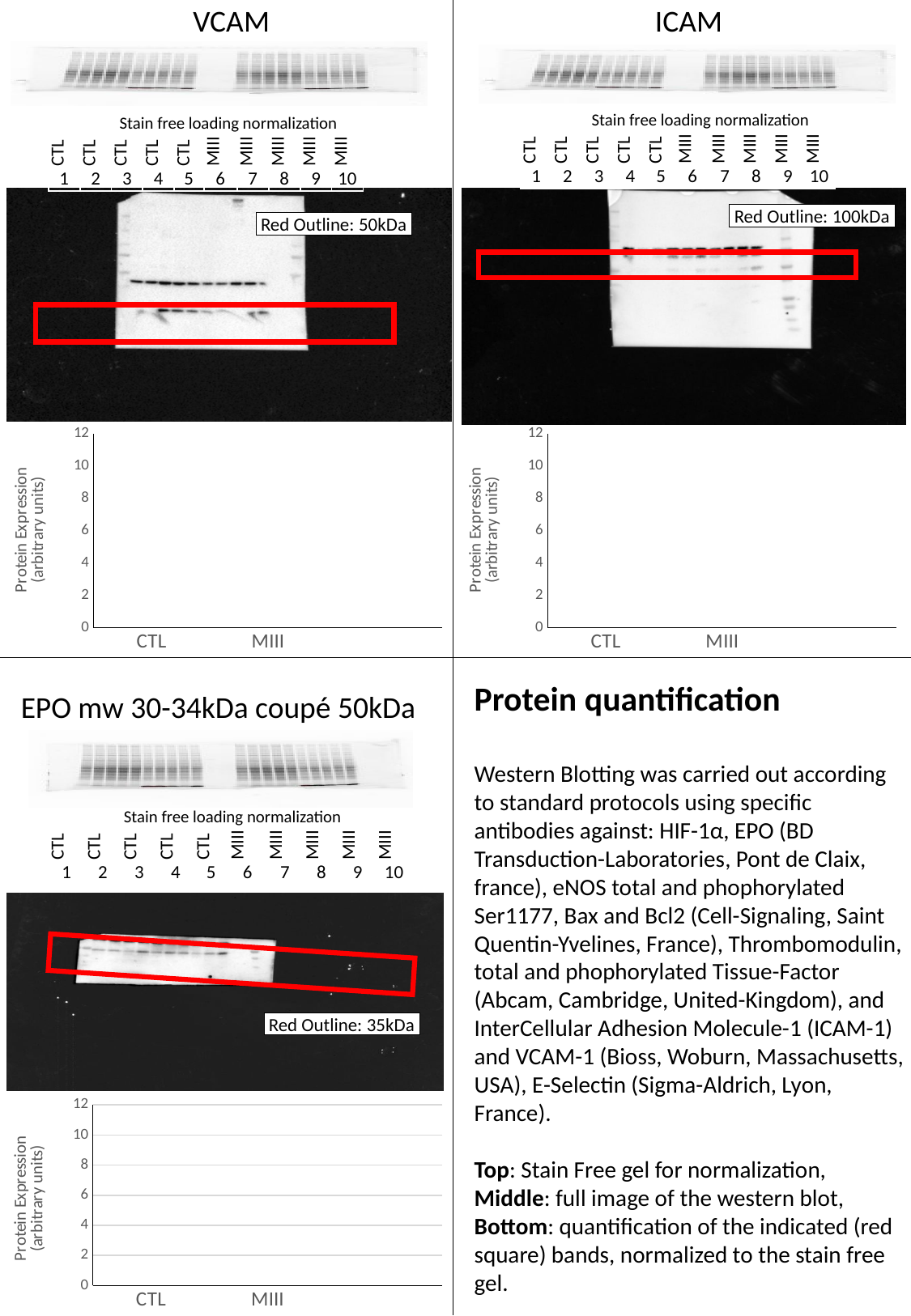

VCAM
ICAM
Stain free loading normalization
Stain free loading normalization
| CTL | CTL | CTL | CTL | CTL | MIII | MIII | MIII | MIII | MIII |
| --- | --- | --- | --- | --- | --- | --- | --- | --- | --- |
| 1 | 2 | 3 | 4 | 5 | 6 | 7 | 8 | 9 | 10 |
| CTL | CTL | CTL | CTL | CTL | MIII | MIII | MIII | MIII | MIII |
| --- | --- | --- | --- | --- | --- | --- | --- | --- | --- |
| 1 | 2 | 3 | 4 | 5 | 6 | 7 | 8 | 9 | 10 |
Red Outline: 100kDa
Red Outline: 50kDa
### Chart
| Category | |
|---|---|
| CTL | 99.94307 |
| MIII | 102.20367200000001 |
### Chart
| Category | |
|---|---|
| CTL | 58.43768 |
| MIII | 96.512882 |Protein quantification
Western Blotting was carried out according to standard protocols using specific antibodies against: HIF-1α, EPO (BD Transduction-Laboratories, Pont de Claix, france), eNOS total and phophorylated Ser1177, Bax and Bcl2 (Cell-Signaling, Saint Quentin-Yvelines, France), Thrombomodulin, total and phophorylated Tissue-Factor (Abcam, Cambridge, United-Kingdom), and InterCellular Adhesion Molecule-1 (ICAM-1) and VCAM-1 (Bioss, Woburn, Massachusetts, USA), E-Selectin (Sigma-Aldrich, Lyon, France).
Top: Stain Free gel for normalization, Middle: full image of the western blot, Bottom: quantification of the indicated (red square) bands, normalized to the stain free gel.
EPO mw 30-34kDa coupé 50kDa
Stain free loading normalization
| CTL | CTL | CTL | CTL | CTL | MIII | MIII | MIII | MIII | MIII |
| --- | --- | --- | --- | --- | --- | --- | --- | --- | --- |
| 1 | 2 | 3 | 4 | 5 | 6 | 7 | 8 | 9 | 10 |
Red Outline: 35kDa
### Chart
| Category | |
|---|---|
| CTL | 124.53680000000001 |
| MIII | 237.07157999999998 |
